# Dry eye disease and tear film assessment through a novel non-invasive ocular surface analyzer: The OSA protocol

**DOI:** 10.3389/fmed.2022.938484

**Published:** 2022-08-10

**Authors:** María Carmen Sánchez-González, Raúl Capote-Puente, Marta-C García-Romera, Concepción De-Hita-Cantalejo, María-José Bautista-Llamas, Carmen Silva-Viguera, José-María Sánchez-González

**Affiliations:** Vision Science Research Group, Vision Sciences of the University of Seville (CIVIUS), Department of Physics of Condensed Matter, Optics Area, University of Seville, Seville, Spain

**Keywords:** ocular surface analyzer, dry eye disease (DED), dry eye syndrome diagnostic, tear film, non-invasive ocular devices

## Abstract

We describe the role of OSA as a new instrument in the study of dry eye, and we recommend a protocol for conducting the tests as well as describe the advantages and disadvantages compared with other instruments. A comparison with other ocular surface devices (Tearscope Plus, Keratograph 5M, anterior-segment ocular coherence tomography, Easy Tear View-Plus, LipiView, IDRA, and LacryDiag) were presented due to manual or automatic procedure and objective or subjective measurements. The purpose of this study was to describe the OSA as new non-invasive dry eye disease diagnostic device. The OSA is a device that can provide accurate, non-invasive and easy-to-use parameters to specifically interpret distinct functions of the tear film. This OSA protocol proposed a lesser to higher non-invasive ocular surface dry eye disease tear film diagnostic methodology. A complete and exhaustive OSA and OSA Plus examination protocol was presented within the subjective questionnaire (Dry Eye Questionnaire 5, DEQ5), limbal and bulbar redness classification (within the Efron grade Scale, interferometry lipid layer thickness (LLT) (according to Guillon pattern), tear meniscus height (manually or automatic), first and mean non-invasive break up time (objective and automatic) and meibomian gland (MG) dysfunction grade and percentage (objective and automatic). The OSA and OSA Plus devices are novel and relevant dry eye disease diagnostic tools; however, the automatization and objectivity of the measurements can be increased in future software or device updates. The new non-invasive devices supposed represent a renewal in the dry eye disease diagnosis and introduce a tendency to replace the classic invasive techniques that supposed less reliability and reproducibility.

## Introduction

Ocular surface pathology is a general term that includes dry eye, with involvement of the cornea, conjunctiva, eyelids, and meibomian glands (MGs). Dry eye is a group of disorders characterized by loss of tear film homeostasis, due to either lipid layer alteration owing to the MGs (evaporative dry eye) or insufficient aqueous tear production (hyposecretory dry eye) leading to tissue damage and inflammation (1).

There are various techniques for measuring and diagnosing dry eye. The most common tests for this diagnosis are invasive and can yield results that differ from the natural properties of the tear, so non-invasive methods would be more appropriate (2). Ocular surface diagnostic tests for dry eye disease should combine high precision, good sensitivity and reproducibility. Among the most commonly used diagnostic devices, Placido method rings have been used in different studies as an alternative to break-up time (BUT) to avoid the use of fluorescein, although they have a weak correlation with other dry eye disease diagnostic measurements (3).

It has been recommended that ocular surface measurements be performed from less invasive to more invasive (4). Such measurements include the use of a questionnaire to collect symptoms (5), evaluation of limbal and bulbar conjunctival hyperemia (6), assessment of tear meniscus (7), study of lipid layer thickness (LLT) and pattern (8), non-invasive tear break-up time (NIBUT) (9) and infrared meibography (10). However, some of the measures used to evaluate dry eye can be influenced by the subjectivity of the examiner.

Among the non-invasive devices for dry eye measurement are Tearscope Plus^®^ (Keeler, Windsor, United Kingdom), Polaris (bon Optic, Lübeck, Germany), EasyTear Viewplus^®^ (EasyTear, Rovereto, Italy), Oculus Keratograph 5M^®^ (Oculus, Arlington, WA, United States) (K5M), LipiView^®^ interferometer (TearScience Inc., Morrisville, NC, United States), IDRA^®^ Ocular Surface Analyzer from SBM System^®^ (Orbassano, Torino, Italy), LacryDiag^®^ Ocular Surface Analyzer (Quantel Medical, Cournon-d’Auvergne, France) and Ocular Surface Analyzer (OSA) from SBM System^®^ (Orbassano, Torino, Italy) (11–13). A summary of the functionalities of the ocular surface devices is presented in Table 1. Regarding Tearscope Plus, the device is attached to the slit lamp, and the measurement is achieved through image analysis software (14). Polaris uses LED light to improve the visibility of both the lipid layer of the tear film and the tear meniscus (15). On the other hand, Oculus Keratograph introduces tear analysis software with an integrated caliper that allows capturing images for a better measurement of the height of the tear meniscus (16). Anterior segment optical coherence tomography (AS-OCT) also allows the measurement of the height of the tear meniscus through integrated software, producing a very high-quality resolution in micrometers. AS-OCT and Keratograph are two comparable methods (17). EasyTear Viewplus^®^ is also attached to the slit lamp, and through white LED lights, it achieves analysis of the lipid layer, NIBUT and tear meniscus; with infrared LEDs, it performs meibography, and the software quantifies the image structures (18). LipiView^®^ allows automated measurements of the lipid layer with nanometer precision. The limitation is that only values greater than 100 nm are displayed (19). IDRA^®^ is attached to the slit lamp to perform the measurement quickly and in a fully automated manner (20). LacryDiag^®^ uses white light in its system to capture images and infrared light for the analysis of the MGs (13). Finally, OSA^®^ is designed to perform dry eye assessment based on the following diagnostic measurements: Dry Eye Questionnaire (DEQ-5), limbal and bulbar conjunctival redness classification, tear meniscus height, LLT interferometry, NIBUT, and meibography gland dysfunction loss percentage.

**TABLE 1:** Ocular surface diagnostics devices comparison.

	Questionnaire	Redness hyperemia	Meniscus	Lipid layer	NIBUT	Meibomian glands
Tearscope plus	−	−	Manual	Guillon pattern	Subjective	−
Polaris	−	−	−	Guillon pattern	Subjective	−
Keratograph 5M	OSDI^a^	R Scan	Manual	Guillon pattern	Objective	Objective
AS-OCT^b^	−	−	Manual	−	−	−
EasyTear ViewPlus	−	−	Manual	Guillon pattern	Subjective	Subjective
LipiView	−	−	Manual	Guillon pattern	−	Subjective
IDRA	SPEED^c^	Efron scale	Manual/automatic	Guillon pattern	Objective	Objective
LacryDiag	−	Efron scale	−	−	Objective	Subjective
OSA (Plus)	DEQ-5^d^	Efron scale	Manual/automatic	Guillon pattern	Objective	Objective

^a^OSDI, Ocular Surface Disease Index.

^b^AS-OCT, Anterior Segment Ocular Coherence Tomography.

^c^SPEED, Standard Patient Evaluation of Dry Eye.

^d^DEQ-5, Dry Eye Questionnaire 5-item.

In the present study, we describe the role of OSA as a new instrument in the study of dry eye, and we recommend a protocol for conducting the tests as well as describe the advantages and disadvantages compared with other instruments.

## Materials and equipment

### Questionnaire

Many questionnaires to analyze and classify symptoms are entered into the software of the instruments for dry eye assessment: Ocular Surface Disease Index (OSDI) in Keratograph 5M (21), Standard Patient Evaluation of Eye Dryness Questionnaire (SPEED) in IDRA (20) and Dry Eye Questionnaire (DEQ-5) in OSA (5). On the contrary, LD (3, 22), LipiView (19, 20), EasyTear Viewplus, Polaris and Tearscope Plus (23, 24) have no questionnaires in their software.

The sensibility and specificity are influenced not only by the number of items in each questionnaire, or the time studied but also by the capacity to classify symptoms. The OSDI is a 12-item questionnaire focusing on dry eye symptoms and their effects in the previous week. In subjects with and without dry eye disease, the OSDI has shown good specificity (0.83) and moderate sensitivity (0.60) (25). The SPEED has eight items to evaluate the frequency and severity of symptoms in the last 3 months. Sensibility and specificity values are 0.90 and 0.80, respectively (26, 27). In the DEQ-5, the symptoms in the past week are analyzed through five questions. This survey has been validated in comparison to the OSDI (Spearman correlation coefficients, *r* = 0.76) (28) and (*r* = 0.65, *p* < 0.0001). The sensitivity is 0.71, and the specificity is 0.83 (29). Thus, any of these three questionnaires could be a good option to analyze dry eye symptoms, although the DEQ-5 might be quicker to use, given the number of items. The advantage that OSA presents with respect to other dry eye analyzers is that the questionnaire has few items and is completed quickly. However, as disadvantages, we find that questionnaires with a greater number of items have greater repeatability.

### Limbal and bulbar redness classification

Regarding the limbal and bulbar redness classifications (LBRC), Keratograph 5M has software (R Scan) to save images and objectively classify them into four degrees ranging from 0 to 3 (30). IDRA, LacryDiag and OSA use subjective procedures, given that the software only shows the image taken and the analysis must be carried out by an observer using a scale (31).

Efron is software widely used to subjectively classify redness in eyes (entered in OSA, IDRA and LacryDiag). The Efron scale has achieved excellent reproducibility (32, 33) and is one of the more accurate scales based on fractal dimension (34). Comparing objective and subjective redness classifications, the highest reproducibility is observed when hyperemia is assessed and scored automatically (6, 30). Among the rest of the ocular surface devices, Tearscope Plus, Polaris, EasyTear Viewplus and LipiView interferometer do not offer a redness analyzer. Therefore, the ideal device has to implement and automatic, objective, non-invasive LBRC assessment integrated into a platform and software within the rest of the ocular surface parameters. The advantage that OSA presents with respect to other dry eye analyzers is that the LBRC is carried out according to the international scale established by Efron. However, as disadvantages, we find that the analysis of redness is subjective while the Keratograph 5M presents a software that performs it objectively and automatically.

### Lipid layer thickness

There are different devices to measure the thickness of the lipid layer, most of which are based on optical interferometry, such as OSA. These devices are Tearscope Plus, EasyTear Viewplus, Polaris, Keratograph 5M, and LipiView. The basic technology in them is the same; the measurement is performed non-invasively by observing the phenomenon of interference fringes, which allows the thickness of the lipid layer secreted by the MGs to be analyzed.

With Tearscope Plus, EasyTear Viewplus and Polaris, the result obtained has a subjective and qualitative component, as the observer compares the image he sees with the same classification that exists for the thickness of the lipid layer in five different categories as described by Guillon (35) (amorphous structure, marbled appearance, wavy appearance, yellow, brown, blue or reddish interference fringes). This same classification allows a quantitative equivalent (from thinner to thicker: < 15 nm–not present, ∼15 nm–open meshwork, ∼30 nm–closed meshwork, ∼30/80 nm–wave, ∼80 nm–amorphous, ∼80/120 nm–color fringes, ∼120/160 nm–abnormal color) used by OSA and IDRA. Keratograph 5M uses four interferometric patterns instead of five 1 = open mesh (13–15 nm); 2 = closed mesh (30–50 nm); 3 = wave (50–80 nm); and 4 = color fringe (90–140 nm). In both devices, the subjectivity of the observer is influential during classification; this type of measurement is considered to be more reliable and repeatable, with less deviation in the results (36–38).

Only LipiView is capable of measuring with nanometer precision (39). It is a non-invasive instrument that takes live digital images of the tear film, measures its lipid component, and assesses LLT using an interference color unit (ICU) score (usual average ≥ 75 score points). Illumination is projected over the lower third of the cornea from a color interference pattern as a result of the specular reflection at the lipid aqueous border. The detected color is related to the device and is shown as an ICU, which is equivalent to nanometers.

Different publications support the reliability of the LLT measurement with LipiView, both in its value as a diagnostic element compared to other devices in which the observer intervenes and in its intra- and interobserver repeatability (19, 20, 40, 41). The advantage that OSA presents with respect to the rest of dry eye analyzers is that the classification of the lipid pattern of the tear film is carried out in accordance with the international scale established by Guillon. However, as disadvantages, we find that the analysis of the lipid thickness is of a qualitative nature, while LipiView presents a software that measures the thickness of the lipid layer quantitatively.

### Tear meniscus height

Several ocular surface devices (EasyTear Viewplus, AS-OCT, Keratograph5 M, LipiView, OSA and IDRA) present the possibility of measuring tear meniscus height, and the acquisition of multiple images is performed non-invasively, as the water content can be accurately evaluated with an integrated caliper along the edge of the lower or superior eyelid. OSA Plus and IDRA are unique devices that automatically and objectively measure the tear meniscus height of the lower lid. Scientific evidence is needed to establish the repeatability and reproducibility of these devices.

The works presented on tear meniscus height are scarce, but they support its repeatability, in both the one carried out in a slit lamp (42) and the one completed with Keratograph 5M, which has a significant correlation with traditional diagnostic tests for dry eye disease (43, 44). Future lines of research should measure the tear meniscus volume instead of the height to estimate the aqueous layer of the tear. The advantage that OSA presents with respect to other dry eye analyzers is that the height of the tear meniscus is measured manually (with OSA) and automatically (with OSA Plus), making it an objective test. In this sense, the rest of the dry eye analyzer devices perform a manual measurement of the height of the tear meniscus.

### Non-invasive break-up time

NIBUT is objectively measured by Keratograph 5M, OSA, IDRA and LacryDiag. These devices record the first alteration of the tear film (FNIBUT) as well as the average BUT for all points of measurement (MNIBUT). Keratograph 5M (45–48) performs the measurement automatically for 24 s, but using OSA (49), IDRA (12, 50, 51) and LacryDiag (13, 52), the clinician manually activates and stops video recording. Keratograph 5M has shown good repeatability and reproducibility in patients with dry eye and healthy controls (43). It is the most commonly utilized instrument in ocular surface studies and is used for the validation of the other devices (11, 13, 36, 53). OSA and LacryDiag measurements of NIBUT are obtained through the detection of distortions in circular rings that are reflected in the tear film using the Placido rings accessory (13). Employing OSA Plus and IDRA, grids can be inserted into the internal cylinder of the device to project structured images onto the surface of the tear film, and the examiner can choose between manual or automatic analysis. In a validation study, IDRA showed good sensitivity and specificity values for NIBUT (12).

NIBUT can be subjectively measured by Tearscope Plus, Polaris and EasyTear Viewplus. These instruments project a grid of equidistant circles of light onto the surface of the eye that are blurred by the tear film rupture. The NIBUT is taken as the time elapsed until the blur of the lines can be observed. Polaris (54), EasyTear Viewplus (55), TS (56–58) and Keratograph 5M produced similar average results relating to NIBUT in the study carried out by Bandlitz et al. (11). Because Keratograph 5M is the only device that performs the NIBUT measurement fully automatically, it is the recommended instrument for the measurement of this parameter. The advantage that OSA presents with respect to the rest of dry eye analyzers is that the measurement of the FNIBUT and MNIBUT is carried out automatically and objectively. Therefore, it is on a par with other dry eye analyzer devices such as the Keratograph 5M and the LacryDiag.

### Meibomian gland dysfunction

Non-contact infrared meibography is a technique used to study MG dysfunction by evaluating MG dropout. The qualification of the degree of MG dropout can be determined subjectively by means of a scale or objectively through software that automatically calculates the relationship between the area of loss of MG and the total area of the eyelid (value ranging from 0 to 100%) (59). Automatic objective measures may be more useful for detecting early gland loss (60).

The non-invasive instruments that can perform the study of MG dysfunction are Keratograph 5M, OSA, IDRA, EasyTear Viewplus, LacryDiag and LipiView. The analysis of meibography with EasyTear Viewplus and LipiView (20, 61, 62) is carried out subjectively by comparing it with a scale. In LacryDiag, the analysis is semiautomatic. The examiner manually delimits the exam area, and the software provides the percentage of MG loss (13). OSA (49) and IDRA (12, 20, 50, 51) have automatic, semiautomatic or manual procedures for analyzing the present and absent gland area and show MG loss in a classification of four degrees: 0–25, 26–50, 51–75, and 76–100%. In the manual procedure, the examiner selects the area in which the MGs are located. In addition, OSA Plus and IDRA perform automatic 3D meibography. Using Keratograph 5M, the analysis can be subjective by comparing the image obtained with a reference scale with four degrees (ranging from 0 to 3) (13, 45, 46) or semiautomatic through the ImageJ software that provides the total area analyzed and the area covered by MGs (47, 60, 63, 64). The advantage that OSA presents with respect to the rest of dry eye analyzers is that the measurement of the MGD percentage is carried out automatically and objectively. Therefore, it represents an improvement over other dry eye analyzer devices such as the Keratograph 5M and the LacryDiag that perform manual or semi-automatic measurement using software.

## The ocular surface analyzer protocol: Methods and anticipated results

**FIGURE 1:**
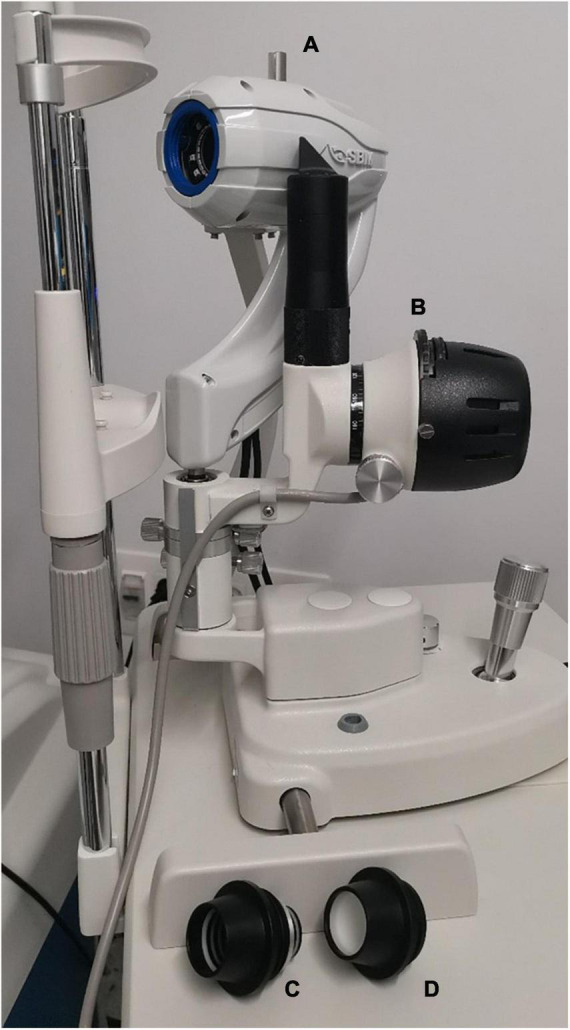
Ocular Surface Analyzer (OSA) device fit in a slit lamp tonometer hall. **(A)** OSA measurement head device. **(B)** Slit lamp illumination system. **(C)** Placido grid measurement cone. **(D)** Plain measurement cone.

Non-invasive tear film analysis is performed with the Integrated Clinical Platform (ICP) within the OSA. The OSA includes a full assessment of the ocular surface through a combination of dry eye disease diagnostic tests. The test allows the quick assessment of the details of the tear film composition, including the lipid, aqueous and mucin layers, in addition to conjunctival redness classification and MG assessment. The instrument is fit in the slit lamp tonometer hall. Regarding the technical data, the image resolution is six megapixels, the acquisition mode is multishot and movie acquisition, the focus can be manual or automatic, and Placido disc and NIBUT grids are available. Furthermore, the color and sensitivity to infrared cameras are accessible, and the light source is an infrared or blue light-emitting diode (LED). An OSA device image was presented in Figure 1.

The OSA protocol examination includes all available non-invasive dry eye disease tests in the device. Temperature and humidity room examination conditions must be stable during all measurements. Illumination of the room should be performed under mesopic conditions. The patient must not wear soft or rigid contact lenses at least 48 h prior to the examination. In addition, no lubricants, eyedrops or make-up should be used before the measurements. Ocular surface tests are taken in alternating fashion between both eyes. Furthermore, between OSA measurement steps, the subjects blink normally within 1 min. Prior to the next measurement, the subject blinks deliberately three full times. The order of the measurements is from minor to major tear film fluctuations in the following order.

### Subjective questionnaire

The questionnaire included in the OSA platform is the DEQ-5 (5, 65–67). It has five questions divided into three blocks: (I) Questions about eye discomfort: (a) During a typical day in the past month, how often did you feel discomfort (from never to constantly) and (b) When your eyes feel discomfort, how intense was the feeling of discomfort at the end of the day, within 2 h of going to bed? (from never have it to very intense). (II) Questions about eye dryness: (a) During a typical day in the past month, how often did your eyes feel dry? (from never to constantly) and (b) When you felt dry, how intense was the feeling of dryness at the end of the day, within 2 h of going to bed? (from never have it to very intense). (III) Question about watery eyes: (a) During a typical day in the past month, how often did your eyes look or feel excessively watery? (from never to constantly).

At the end of the questionnaire, the OSA platform summarizes the results, with scores ranging from 0 to 4 for questions I-a, II-a and III and scores ranging from 0 to 5 for questions I-b and II-b. The total possible score in this questionnaire is 22 points. Chalmers et al. (5) described mean healthy population results of 2.7 ± 3.2 points within a clinical difference to detect six points (68) (based on the variation between severity classification) (5).

### Limbal and bulbar redness classification

**FIGURE 2:**
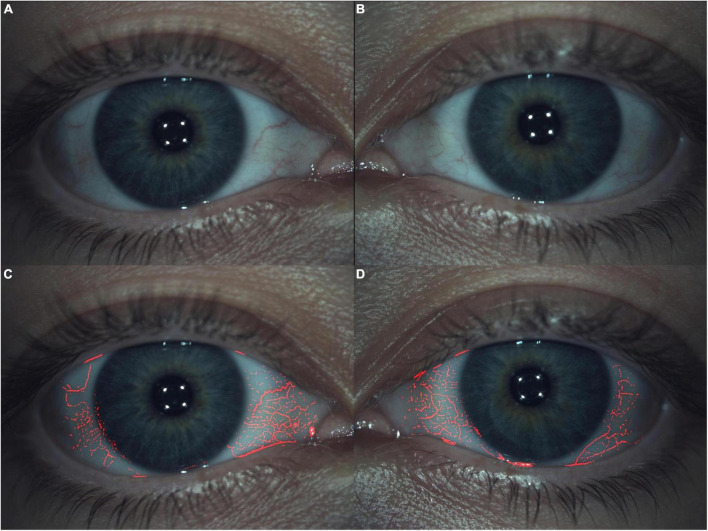
Limbal and bulbar redness classification. All presented images are Grade 1 within the Efron Scale. **(A,B)** Right and left eye, respectively, with blood vessels fluidity of conjunctiva switch off. **(C,D)** The same right and left eye, respectively, with blood vessels fluidity of conjunctiva switch on.

The LBRC was detected within the blood vessel fluidity of the conjunctiva to evaluate the redness degree with the Efron (69) Scale (0 = normal, 1 = trace, 2 = mild, 3 = moderate and 4 = severe). For this measurement, no cone was placed on the device. A central picture must be taken to assess limbal conjunctival redness (Figure 2). Therefore, a nasal and temporal picture must be taken to assess bulbar conjunctival redness (Figure 1). Efron (69) and Wu et al. (30) did not report mean healthy population values, although they established clinically normal as grade 0–1. The clinical difference to detect is 0.5 grading (68).

### Lipid layer thickness

**FIGURE 3:**
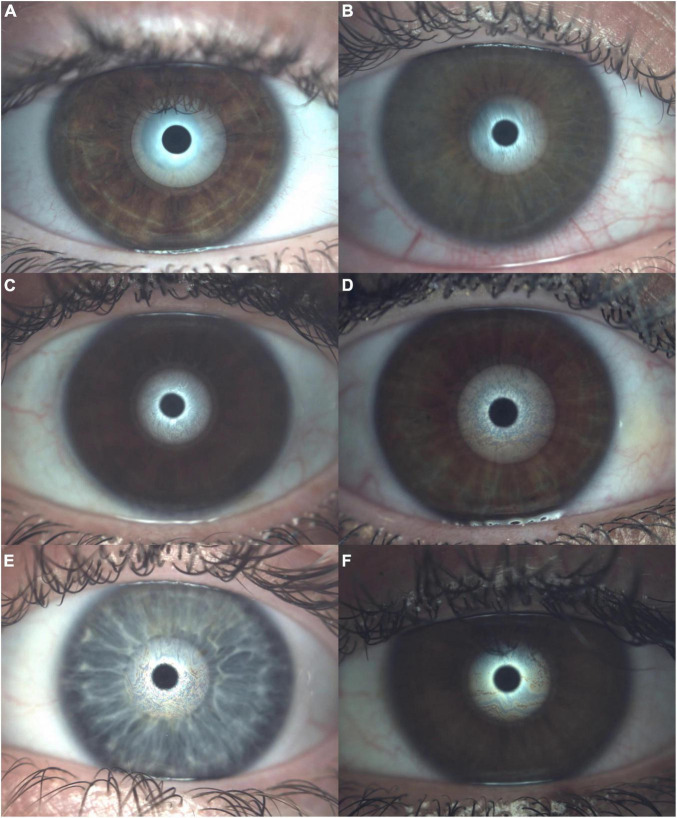
Lipid layer thickness assessment within the optic interferometer. **(A)** No lipid present (<15 nm of lipid thickness). **(B)** Open meshwork pattern (∼15 nm of lipid thickness). **(C)** Close meshwork pattern (∼30 nm of lipid thickness). **(D)** Wave pattern (∼30/80 nm of lipid thickness). **(E)** Amorphous pattern (∼80 nm of lipid thickness). **(F)** Color fringes pattern (∼80/120 nm of lipid thickness) and no patient achieved abnormal color (∼120/160 nm of lipid thickness).

At this point, the quality of the tear film lipid was assessed. The LLT evaluation was performed with optic interferometry. Furthermore, the evaluation of the quantity of the lipid layer was classified into seven different pattern categories defined by Guillon (35). For this measurement, a plain cone is placed on the device. The patient must blink normally during an approximately 10-s video recording. Later, the video is compared with the seven videos to match the exact lipid layer pattern (Figure 3).

### Tear meniscus height

**FIGURE 4:**
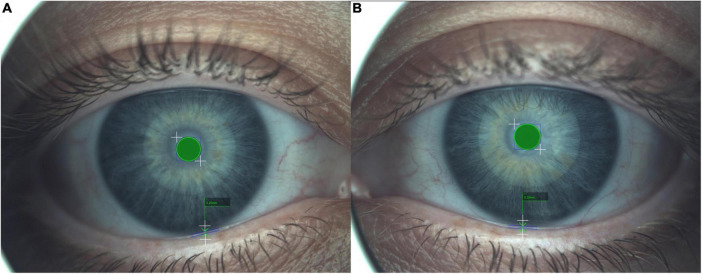
Tear meniscus height (TMH) measured with the caliper. The central green circle represents a standard measure of reference to calculate the TMH. **(A,B)** Images represent the right and left eye, respectively. A result ≤0.20 mm implies an abnormal TMH and >0.20 mm suppose a within the norm TMH.

The TMH test evaluates the aqueous layer quantity within a millimeter caliper (≤ 0.20 mm–abnormal and > 0.20 mm–normal). For this measurement, the plain cone is placed on the device. The picture consists of a central capture of the tear meniscus focalized in the center of the green square (Figure 4). Later, the millimeter caliper is placed at the start and end of the tear meniscus, and the height is obtained. Multiple measurements can be performed as well as nasal or temporal TMH. Mean healthy population results were presented by several authors. Nichols et al. (42) reported 0.29 ± 0.13 mm (measured with a slit lamp), Wei et al. (44) reported 0.29 ± 0.04 mm (measured with Keratograph 4), Tian et al. (43) reported 0.27 ± 0.12 mm (measured with Keratograph 5M), Li et al. (70) reported 0.19 ± 0.02 mm (measured with ocular coherence tomography, OCT) and Wang et al. (71) reported 0.34 ± 0.15 mm (measured with OCT). The minimal clinical difference to detect was set at 0.1 mm (68).

### Non-invasive break-up time

**FIGURE 5:**
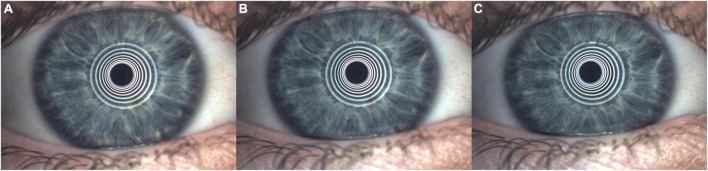
Non-Invasive Break-Up Time (NIBUT). **(A)** Placid disk rings reflected on tear film just after initial double deliberate blinks. **(B)** First Placido rings deformation (difficult to see visually by a human) this moment automated establishes the first non-invasive break-up time (FNIBUT). **(C)** Mean and general Placido rings deformation (difficult to see visually by a human) this moment automated establishes the mean non-invasive break-up time (MNIBUT).

Regarding this measurement, the tear film mucin layer quantity is assessed. The FNIBUT and MNIBUT are evaluated with a special grid cone, which evaluates the tear film break in seconds. The Placido cone is set for this test. The patient must deliberately blink two times; after this, the video recording starts and stops at the first involuntary blink. The device auto analyzes the measurement and reports the first point of the blur grid as the FNIBUT and the generalized tear film BUT as the MNIBUT (Figure 5). Mean healthy population results were established by Nichols et al. (58) 11.2 ± 6.8 s (measured with Tearscope Plus) and Tian et al. (43) 10.4 ± 4.2 s (measured with Keratograph 5M). The minimal clinical difference to detect was set at 5 s (68).

### Meibomian glands dysfunction

**FIGURE 6:**
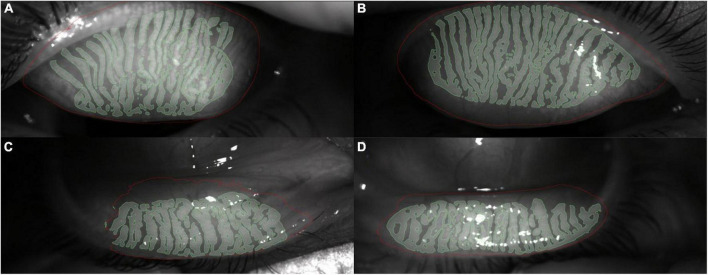
Meibomian gland pattern and dysfunction measured with an infrared non-contact camera. All images were real, and the green zone automatic or manual establishes glands presence. **(A)** Right eye upper eyelid real meibomian gland pattern. **(B)** Left eye upper eyelid real meibomian gland pattern. **(C)** Right eye low eyelid real meibomian gland pattern. **(D)** Left eye low eyelid real meibomian gland pattern.

**FIGURE 7:**
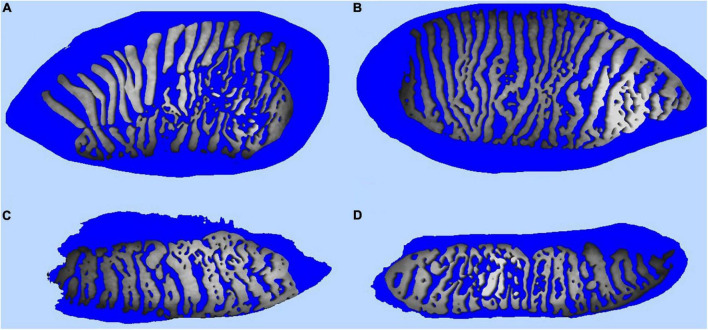
Simulated 3D meibomian gland pattern performed with the intranet software of the Integrated Clinical Platform (ICP) within the Ocular Surface Analyzer (OSA) from SBM System ^®^ (Orbassano, Torino, Italy). **(A)** Simulated 3D right eye upper eyelid real meibomian gland pattern. **(B)** Simulated 3D left eye upper eyelid real meibomian gland pattern. **(C)** Simulated 3D right eye low eyelid real meibomian gland pattern. **(D)** Simulated 3D left eye low eyelid real meibomian gland pattern.

The MG dysfunction percentage us measured with an infrared non-contact camera that evaluates the upper and lower lid after everting it with a swab. For this measurement, no cone is placed on the device. MG pictures of the upper and lower eyelids must be captured inside the green square. After the catch, MG assessment can be performed automatically or manually (Figure 6). In addition, a combination of both methods can be performed with the semiautomated method that allows the addition or removal of non-detected MGs manually. The MG dysfunction percentage can be classified into four degrees: ∼0%–Grade 0, < 25%–Grade 1, 26–50%–Grade 2, 51–75%–Grade 3 and > 75%–Grade 4 (72, 73). The device permit to perform a simulated or real (with OSA Plus) 3D MG pattern (Figure 7).

## Future research lines and limitations

New emerging lines of research are focused on the search for identifiers that allow us to recognize biomarkers of the effects of the ocular surface in a more objective, automated and minimally invasive way. To enhance the field, the development of new algorithmic calculations and the incorporation of software for data analysis, such big data and machine learning, will allow us to recognize, detect and classify more accurately the different values, including the interrelations between them, in an automated way with different parameters (74). Independent and dissociated observation of the tear film, inclusion of palpebral parameters and analysis of proinflammatory factors without the need for invasive, expensive, rapid or invited tests are potential future directions that should be analyzed (75, 76).

Future researchers should consider that the intensity of illumination produced by these instruments in their measurements can cause an increase in the blink rate and reflex tearing (77). Therefore, the main limitations found are the lack of objectivity and automation in the measures conducted, absence of correlations between existing tests and lack of extrapolation to other similar systems. However, the lack of intra- and interobserver repeatability in some of the measurement tools due to the interaction of an observer limits neutrality and increases biases, which impact the validity of the results. Within the limitations of this study, an accuracy and repeatability research is needed to validate this ocular surface device.

## Conclusion

The OSA is a device that can provide accurate, non-invasive and easy-to-use parameters to specifically interpret distinct functions of the tear film. The use of variables and subsequent analysis of results can generate relevant information for the management of clinical diagnoses. The OSA and OSA Plus devices are novel and relevant dry eye disease diagnostic tools; however, the automatization and objectivity of the measurements can be increased in future software or device updates.

## Data availability statement

The original contributions presented in this study are included in the article/supplementary material, further inquiries can be directed to the corresponding author.

## Author contributions

MS-G, RC-P, M-CG-R, CD-H-C, M-JB-L, CS-V, and J-MS-G: conceptualization, methodology, writing—original draft preparation, writing—review and editing and supervision. All authors read and agreed to the published version of the manuscript.
